# Multi-Sensory Data Fusion in Terms of UAV Detection in 3D Space

**DOI:** 10.3390/s22124323

**Published:** 2022-06-07

**Authors:** Janusz Dudczyk, Roman Czyba, Krzysztof Skrzypczyk

**Affiliations:** 1 Institute of Information Technology and Technical Sciences, Stefan Batory State University, 96-100 Skierniewice, Poland; 2 Department of Automatic Control and Robotics, Silesian University of Technology, 44-100 Gliwice, Poland; roman.czyba@polsl.pl (R.C.); krzysztof.skrzypczyk@polsl.pl (K.S.)

**Keywords:** UAV, anti-drone system, data fusion, drone detection, identification, recognition, sensing technologies, tracking algorithm

## Abstract

The paper focuses on the problem of detecting unmanned aerial vehicles that violate restricted airspace. The main purpose of the research is to develop an algorithm that enables the detection, identification and recognition in 3D space of a UAV violating restricted airspace. The proposed method consists of multi-sensory data fusion and is based on conditional complementary filtration and multi-stage clustering. On the basis of the review of the available UAV detection technologies, three sensory systems classified into the groups of passive and active methods are selected. The UAV detection algorithm is developed on the basis of data collected during field tests under real conditions, from three sensors: a radio system, an ADS-B transponder and a radar equipped with four antenna arrays. The efficiency of the proposed solution was tested on the basis of rapid prototyping in the MATLAB simulation environment with the use of data from the real sensory system obtained during controlled UAV flights. The obtained results of UAV detections confirmed the effectiveness of the proposed method and theoretical expectations.

## 1. Introduction

During the last two decades, unmanned aerial vehicles have experienced enormous development [1,2]. Since unmanned aerial vehicles (UAVs) were released for general civil use, the number of incidents involving them have been constantly increasing. Unfortunately, the threats they pose may endanger public and personal safety.

This article comprehensively raises the issue of anti-drone systems technology. The current state of knowledge in this area is presented, as well as an overview of the existing market solutions in the field of anti-drone systems that enable counteracting UAVs [3,4,5,6,7]. Radar, visual, acoustic and radio technologies that are used for UAV detection are characterised. The fundamental research problem raised in the article concerns the data fusion from the AeroScope radio system, EchoGuard radar equipped with four antenna sets and ADS-B in order to develop a UAV detection algorithm in 3D space. The article elaborates on the process of pre-processing data from the afore-mentioned sensors, the data synchronisation process and radar data fusions in order to develop a drone detection algorithm. The algorithm was tested on actual data.

In general, the main goal of the paper was to develop an algorithm based on the data fusion from various sensors, enabling the detection, identification and recognition in 3D space of a UAV violating restricted airspace.

The structure of the paper is as follows. First, a brief review of anti-drone systems and a wide range of UAV detection technologies is provided. Then, the concept of the test procedure in real-word conditions, in the presence of both single and multiple drones, is presented. Section 5 contains a description of selected sensors along with data analysis, which has a significant impact on the choice of data fusion method and the development of an algorithm. Section 6 presents the data fusion algorithm along with a description of the individual stages of system design. Finally, Section 7 presents the results of UAVs detection, performed on the data of the actual experiment. The conclusions are discussed in the last section.

## 2. Review of Anti-Drone Systems

Currently, there are numerous solutions in the field of anti-drone systems available on the market. Their main task is to counteract UAVs by detecting and combating them using kinetic methods or energy directed at the UAV in the form of an electromagnetic pulse [8,9]. The most advanced technical solutions use full azimuth coverage of 360° and hemispherical elevation coverage in the range of −45° to 225°. The accuracy of computing the azimuth resolution for these types of systems can even reach the value of ±0.5°, and the accuracy of computing the elevation is ±0.7°. Other competitive systems of this type are available on the market, most often used for limited spatial coverage, both in azimuth and elevation. An example is the AARONIA system based on the AARTOS detector (RF detector), which provides an elevation coverage of 10°, and the SpotterRF system (3D-500 Radar), which provides an elevation coverage of 90°. In addition to the above-mentioned systems that are approved for operation in urban areas, there are also systems that, due to their emission parameters, are not approved for urban usage. These include the Blighter (A400 Radar), ROBIN (ELVIRA), ELTA (ELM2026) and Echodyne (EchoGuard) systems, which not only are not approved for urban usage, but also allow you to only cover the space of: 180° in azimuth and 20° in elevation, 360° in azimuth and 60° in elevation, 90° in azimuth and 60° in elevation and 120° in azimuth and 80° in elevation, respectively. In the latest technical solutions, a single radar sensor is able to provide complete hemispherical spatial coverage and semi-spherical elevation coverage, so there is no need to integrate several RF sensors. This approach minimises mutual electromagnetic interference and at the same time enables the efficient use of the allocated spectrum resources. Furthermore, due to the use of a single radar sensor, the weight of the anti-drone system is significantly minimised. An example is the RS800 solution (by ARTsys360), the weight of which is approx. 5 kg, which is incomparably smaller than other systems operating in urban areas, such as SpotterRF or AARONIA, whose weights are approx. 6 kg and 30 kg, respectively. A highly crucial parameter of anti-drone systems is the detection range. This parameter largely depends on the radar cross section (RCS) of the UAV and the speed at which the recognised UAV is moving. Furthermore, a UAV radar cross-section signature may be a highly efficient distinguishing feature in the process of drone detection and classification as shown in [10]. On average, the detection range of drones for this type of systems ranges from 800 m to 3500 m, moving at a speed of approx. 40 m/s. The exemplary EchoGuard radar by Echodyne is able to detect drones at a distance of 900 m, people at a distance of 2200 m and vehicles at a distance of 3500 m. The accuracy of detection is also a crucial parameter of anti-drone systems, the value of which should be as high as possible, especially when detecting and tracking quickly manoeuvring objects in low-altitude urban areas [11]. In addition to technical parameters, it is also worth mentioning the functional parameters of anti-drone systems, such as software-defined functionality or sensory multi-functionality. The software-defined functionality enables additional functions of the anti-drone system, such as automatic self-calibration, dynamic detection and frequency allocation, dynamic disturbance detection, the automatic launch of anti-disturbance modes, remote system operation and configuration and software updates [12]. The sensory multi-functionality of anti-drone systems is based on the application of multisensory sensor fusion (radar, optical, thermal imaging) enabling the detection and tracking of various types of objects, i.e., drones, people and vehicles in full azimuth and hemispheric coverage, and feature extraction for the classification process of detected objects [13].

For this reason, the data fusion process is a relevant technical and functional parameter that directly affects the effectiveness of UAV detection, recognition and identification by the anti-drone system. Therefore, a modern anti-drone system is a heterogeneous platform on which various sensors and effectors are installed. Only in this way is a modern anti-drone system able to effectively detect, recognise, classify and incapacitate UAVs. Commercial anti-drone systems on the market feature minimal sensory fusion. The mentioned systems are not optimised in terms of the integration of radar, optical or thermal imaging sensors on one platform. This leads to their functional degradation and selective use in the object detection process, while minimising the effectiveness and probability of object detection. At the moment, the analysed systems presented in Section 2 do not have the characteristics of the so-called ‘data fusion’. Also, commercial solutions do not provide these features, contrary to the method proposed by the authors of this article. To conclude, it can be said that most of the anti-drone systems are dedicated to single purposes that prevent the fusion of many different sensors. By optimising the transmitted waveform and automatic frequency allocation to avoid mutual interference, the ability to transmit detected objects in order to increase the probability of detection and minimise false alarms and the possibility of fusing several sensors on a single display in the GUI, the synergistic coexistence of the anti-drone system is ensured.

## 3. UAV Detection Technologies

The methods of detecting unmanned aerial vehicle (UAV) interference can essentially be divided into passive and active methods. In [14], the authors present a comprehensive survey on anti-drone systems and anti-drone system analysis, investigating a wide range of anti-drone technologies and deriving proper system models for reliable drone defence. Each of these methods has its own typical advantages and disadvantages. The most relevant methods and their features are characterised below.

The main advantage of all passive methods is their undetectability by an intruder. This is highly important if you plan to detect and monitor the activity of an intruder in order to identify his intentions. Most passive methods are usually less capable of detecting threats, although this is not always the case. Passive methods are based on receiving signals sent by the drone in different frequency bands (it can be a radio, acoustic or optical signal). There are also methods of detecting magnetic anomalies caused by a moving unmanned vehicle, which by its nature causes a local disturbance of the magnetic field. Observation with the application of an optical camera system using visible light technology or thermal imaging is also a highly effective method. In general, optical observation systems are capable of detecting UAVs at a certain distance. This task is performed with the use of specialised imaging cameras and a mount that allows you to observe the entire protected area. As far as the automatic observation method is concerned, it most often uses image analysis algorithms that are based on the changes in the observed image. This allows moving objects to be distinguished in relation to the stationary background. Initial detection is followed by classification based on matching the observed image to the image pattern. This method requires using very high-resolution cameras to ensure that the image is scanned at a distance of several hundred metres from the camera. The required resolution increases with the square of the distance. In [15], the authors show how the performance of a UAV detection and tracking concept based on acousto-optical technology can be powerfully increased through active imaging. Of course, high resolution requires enormous computing power of the image analyser in order to be able to detect, classify and possibly identify the object in real time. In case of performing in the dark, a system of thermal imaging cameras is used. The advantage of this solution is the fact that the drone usually leaves a noticeable thermal trace during the flight due to the high power generated by its automation. In [16], the authors examined the CNN model that is suitable for visible camera-based drone identification. One of the passive methods of UAV detection is the acoustic method, which relies on tracking the object through listening and sensing and then analysing the sound. This method has been known practically since the beginning of aviation and was already used during World Wars I and II. Using a multi-microphone system, it is possible to pinpoint the direction with an accuracy of several dozen degrees. By using signal analysis with the application of appropriate patterns, it is possible to identify, with high probability, the sound emitted by the drone, which is quite distinctive. A significant drawback of the mentioned method is the influence of ambient sounds (the so-called acoustic background), which has a negative impact on the detection, classification and identification of UAVs. In addition, the strength and direction of the wind have a highly crucial bearing on the range of the acoustic method. Due to the highly limited range resulting from the high attenuation of the acoustic wave in the space of its propagation, acoustic waves are used sporadically for UAV detection. Another passive method is radio-electronic spectrum monitoring, which allows the source of the radio signal to be detected and located. When using this method to detect UAVs, it is necessary to know the radio band that is used to communicate with the drone and the signal structure, allowing for the approximate identification and possible exclusion of irrelevant signal sources. An antenna system with a dedicated receiver is required to detect such a radio-electronic signal. To determine the direction from which the signal is transmitted, it is necessary to provide a directional antenna or an array of antennas providing information to the multi-channel data analyser. This method can be used to determine the direction from which the signal is transmitted, but to pinpoint a specific position (dislocation), at least two such tracking units are required to pinpoint the position of the source by using the triangulation method [17]. Another desirable feature is the ability to identify the radio signal sent both by the drone and the operator itself in order to locate the position of said operator of the unmanned aerial vehicle. The ability to read the transmitted information allows you to obtain telemetry data, for instance, the data about the GPS position. This is possible with standard drones purchased on the market that do not use special encryption. A potential disadvantage of the system is the problem of the identification and interception of radio data in the case of specially developed non-standard structures, other than the so-called ‘off-the-shelf technology’ (COTS—commercial off-the-shelf). Another problem is the use of the drone structure for the so-called ‘silent flight’. In this mode, the UAV turns off the two-way radio communication immediately after take-off and can only receive the signal from the operator (uplink). Then it is impossible to detect it in the standard radio band, and the drone flies along the programmed route and lands at a predetermined location.

Active methods use the signal they send to detect and locate unmanned aerial vehicles. Most often it is a radio (or radar) signal in the form of a directional electromagnetic beam. The afore-mentioned signal reflecting off the surface of an object or target is detected [18]. Knowing the delay of the reflected signal, the distance can be determined. The method is effective for purposes several times greater than the wavelength used in the device. Due to the fact that most often they are centimetre waves or shorter, it is possible to detect drone-type objects. The disadvantage of active methods is the easy detection of the ‘pinpoint’ attempt. In terms of operational activities, this enables an intruder to attempt to withdraw from the intended activities or to hide. Active UAV detection systems operating in the radio band are not dependent on the time of the day or weather conditions. They are widely used for detecting and locating sources of electromagnetic waves, and in case of an active radar beam, they are able to detect even a small object in the protected zone. In [19], the authors design a drone detection mechanism using the RF control signal exchanged between the drone and its remote controller. Fundamental techniques based on radar detection work very well, but they may often be insufficient when confronted with very small UAVs [20]. There are several studies on the analysis of radio signals emitted by UAVs and their controllers. The authors of [21] examine the distinctive features of the radio spectrum for some of the most popular UAV systems and propose an algorithm for detecting the presence of UAVs in the analysed radio spectrum. In [22], the authors propose a per-drone iterated algorithm that optimises drone-cell deployments for different drone-cell numbers and prevents the drawbacks of the pure particle swarm optimisation-based algorithm. The commercial UAV market is developing in an extremely swift manner and new models are based on newer and newer technologies. Therefore, it is vital to update the signature databases for given models, regardless of the selected detection method. Nevertheless, the radio analysis approach seems to have the lowest variability. Currently, the most popular and the most flexible tool for radio spectrum analysis is software defined radio (SDR) platforms. These are software-tunable radio system platforms that allow for signal processing in digital form. This process is mainly based on the conversion of the recording of the radio signal from the time domain to the frequency domain by means of the Fourier transform and its appropriate analysis [23,24].

### Active Methods of Disrupting and Directly Interfering with the UAV

Another way to prevent the drone from undesirable trespassing directly into the safety zone is active interference. This can be executed by disrupting the GPS signal that is necessary for the aircraft positioning and navigation, and also by disrupting or preventing the signal transmission between the operator and the aircraft. Jammers are used for this purpose. They can operate in the band of a specific device or system (e.g., GPS signal) or in a wideband, e.g., to interfere with data transmission. This type of device is most often equipped with a directional antenna that sends a beam aimed at the UAV that needs to be disabled. Sector or omnidirectional antennas are also used to operate in all directions. This solution is used in the defence of a large sector, when it is difficult to locate the target of the attack and precisely pinpoint the direction the UAV is attacking from. The methods of direct interference include active defence through the so-called ‘kinetic’ attack, which may take the form of firing dismantling missiles at the UAV (whose individual parts, after dismantling, hang on Kevlar lines and get entangled with the UAV rotors) or throwing a neutralising net at the hostile UAV. These types of methods can only be used to a limited extent due to the safety of the people in the vicinity. All the detection methods used, along with the sensors and the entire measurement infrastructure, must be connected to an IT system that supervises all activities. It is necessary to secure and organise the work of security services [25]. The structure of the system must enable data to be received from all sensors and security systems, archived and presented in order for appropriate actions to be taken by the system staff. In the case of large objects, it will usually be a distributed multi-station system with several levels of decision-making, both in the context of extracting the signatures of processed signals and building constant vectors (patterns) in the database for further recognition and identification [26].

In the next part of the paper, we will focus on the detection of UAVs that violate the restricted airspace, and not on methods of disrupting and directly interfering with UAVs. Our considerations concern the development of multisensory data fusion, which allows for the precise detection, identification and recognition in 3D space of UAVs or UAV formations with the distinction of individual platforms. Therefore, the review of anti-drone systems presented in Section 2 made it possible to assess the current state of the art in the studied area, while the available UAV detection technologies presented in Section 3, in particular in the field of sensory systems, allowed for the selection of specific sensors that are the subject of research in the next part of the paper.

## 4. Data Acquisition during Operational Activities

The main purpose of this operational procedure is to acquire data from a real sensory system operating in real conditions similar to the future operation of the system. In order to recreate the scenario of a monitored airspace violation, archiving necessary data is required, such as:

- Test start time;
- UAV take-off time;
- Time of violation of the observed airspace;
- Flight path (spatial coordinates associated with recording time);
- Flight parameters.

**Figure 1:**
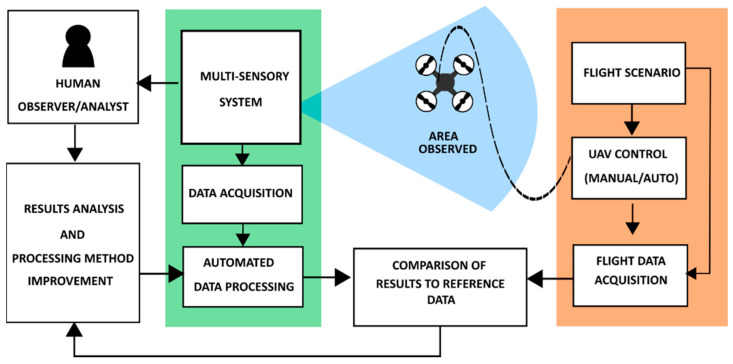
The concept of the system’s tests in realistic conditions.

Figure 1 illustrates the concept of the test procedure. The flight scenario was defined so that the flight trajectory was inside the observation zone.

The data acquisition process took place in real conditions during operational activities under the supervision of qualified operators using unmanned aerial vehicles:

- DJI Matrice 600 with an ADS-B transponder on board;
- DJI Mavic 2.

On the basis of the review of the available UAV detection technologies carried out in the previous section, three sensory systems classified into the group of passive and active methods were selected. At this stage, a holistic approach was applied and the sensors were selected in order to obtain complementarity of the data. At the sensor selection stage, the acoustic method based on tracking and analysing the sound trace emitted by a flying object was abandoned. A significant disadvantage of the method mentioned above is the influence of ambient sounds (the so-called acoustic background), which has a negative impact on the detection, classification and identification of UAVs. Another disadvantage is the strength and direction of the wind, which have a significant impact on the range of the acoustic method. 

Therefore, taking into account the above, the following sensors were selected, from which data were collected during the tests:

- ADS-B transponder;
- DJI AeroScope (the notation ‘AEROSCOPE’ and ‘AeroScope’ will be used interchangeably hereafter) radio system for tracking radio communication between the UAV and the operator;
- EchoGuard radar equipped with four antenna arrays covering a full angle of 360° horizontally and ±40° elevation (will be referred to as ‘radar’ hereafter).

As a result of the tests performed, three data sets were obtained each time, respectively, for each of the sensors. Further on in the paper, the symbols used in the results tables will be explained and the time plots of selected quantities will be presented in a graphical form.

## 5. Sensory Data Analysis

The sensors used in the system under construction determine the use of the following methods to develop effective data fusion algorithms ensuring UAV detection in a controlled airspace:

- Methods based on the ADS-B system;
- Passive radiolocation methods (RF—radio frequency);
- Active radiolocation methods (radars).

### 5.1. ADS-B Transponder

According to the amendment to the aviation law from 31 December 2020, each UAV should be equipped with an ADS-B transponder. The purpose of such an operation is to integrate the UAV with the controlled airspace in which no aircraft can move freely without being visible on the radar by flight controllers and other aircraft, e.g., a passenger equipped with a TCAS (Traffic Alert and Collision Avoidance System)—a collision warning and avoidance system that responds to signals from ADS-B transponders.

The system is event-based and provides data of all the aircraft currently in the airspace and within the range of the ADS-B receiver. Data are contained in an array of 39 columns. Data logged by ADS-B receiver indicate the presence of various aircraft types, classified according to the ICAO classification (International Civil Aviation Organization):

- 0 = unidentified (no information on aircraft type);
- 2 = small aircraft (from 15,500 to 75,000 lb);
- 3 = large aircraft (from 75,000 to 300,000 lb);
- 5 = heavy > 300,000 lb;
- 14 = UAV (unmanned aerial vehicle).

Therefore, data analysis requires a preliminary filtration, narrowing down further considerations to unmanned aerial vehicles only. For the purposes of sensory information analysis and the synthesis of the data fusion algorithm, the following vector of measurement values describing the current state of the system was adopted:

(1) $$X_{1}=\left[\;ICAO,\;lat_{1},\;long_{1},\;h,\;y,\;V_{xy},V_{z},\;ET\right]$$

where:

*ICAO* is the aircraft type code;

*lat*_1_ is the latitude;

*long*_1_ is the longitude;

*h* is the altitude;

y is the heading;

$V_{xy}$ is the horizontal velocity;

$V_{z}$ is the vertical velocity;

*ET* is the category of the aircraft emitting the signal.

The data obtained from the ADS-B transponder are reliable on the condition that the UAV is equipped with such a device. However, the problem is the mass of the ADS-B transponder. Even miniaturised devices are not light enough to be lifted by drones weighing less than 2 kg, and these currently fly the most in the airspace. Accordingly, as of today, there is no guarantee that all drones will be equipped with an ADS-B transponder, so this article proposes a fusion of data from several different sensors.

### 5.2. AeroScope Radio System

The essence of the method is the detection of the RF radio communication signal between the UAV and the ground operator. The flying platform communicates with the controller in a specified frequency band. Once this frequency band has been identified, there is a high probability that a UAV is within the detection range.

The system is event-based and provides pre-processed data in the form of a table with 19 columns, each of which contains temporal data of a specific physical quantity. For the purposes of further analysis of the sensory information and the synthesis of the data fusion algorithm, the following vector of measurement quantities describing the current state of the system has been assumed:

(2) $$X_{2}=\left[V,\;lat_{2},\;long_{2},d,\;h,\;y,\;DT,\;Did\right]$$

where:

*V* is the flight velocity;

*lat*_2_ is the latitude;

*long*_2_ is the longitude;

*d* is the distance of the UAV from the sensory system;

*h* is the altitude;

y is the heading;

*DT* is the UAV type;

*Did* is the UAV identifier.

Based on the obtained flight logs, the time diagrams of basic physical quantities and the reconstructed UAV flight altitude are presented below.

**Figure 2:**
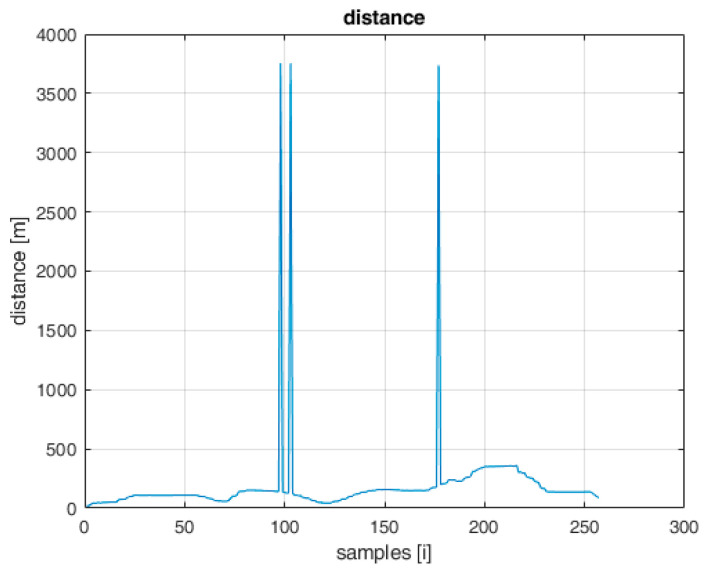
Distance to the UAV measured by AeroScope system.

**Figure 3:**
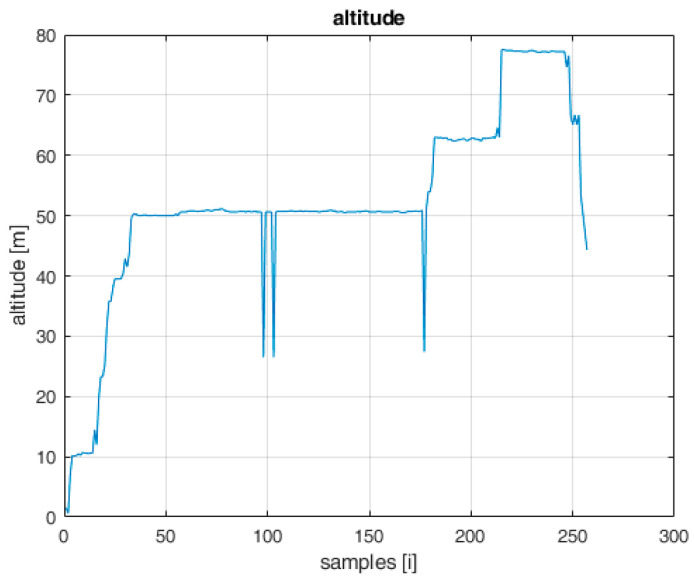
UAV flight altitude broadcasted by AeroScope.

On the graph showing the changes in the distance of the UAV in relation to the AeroScope receiver (Figure 2) and the graph showing the flight altitude (Figure 3), there are noticeable disturbances in the form of large deviations from the regular flight path. Near the 100th and 175th sample, there are visible abrupt changes in distances of a large value, which are not realistic to achieve during the flight with a standard UAV. It can be concluded that these are disturbances that need to be filtered in the step of signal processing. The solution to this problem has been widely discussed in [27].

### 5.3. EchoGuard Radar

The radar system is equipped with four antenna assemblies. The scanning range of a single radar/antenna is 120° in azimuth (±60°) and 80° in elevation (±40°). The antennas are placed every 90° covering the entire area of 360° in azimuth. Taking into account the scanning range of a single antenna of 120°, common scanning areas for adjacent antennas appear.

The system provides data in the form of a table containing 24 columns, each of which is the time data of individual state variables. For the purposes of further analysis of the sensory information and the synthesis of the data fusion algorithm, the following vector of measurement quantities describing the current state of the system has been assumed:

(3) $$X_{3}=\left[\;P_{\mathrm{UAV}},\;CL,\;RCS,\;az,\;el,\;R,\;x,\;y,\;z,\;V_{x},\;V_{y},\;V_{z}\right]$$

where:

*P*_UAV_ is the probability of UAV detection;

*CL* is the confidence level;

*RCS* is the radar cross-section;

*az* is the estimated azimuth;

*el* is the estimated elevation;

*R* is the estimated distance between the UAV and radar;

*x*, *y* and *z* are the estimated UAV coordinates relative to the radar (in the Cartesian coordinate system);

$V_{x},\;V_{y}\;\mathrm{and}\;V_{z}$ are the velocity components of the UAV in the *x*, *y* and *z* axes, respectively.

**Figure 4:**
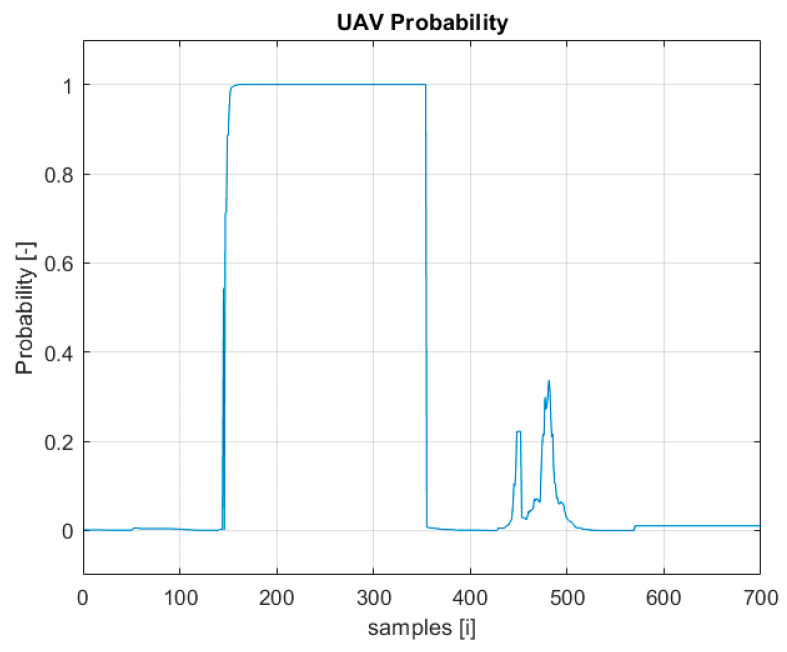
Probability of UAV detection plot.

**Figure 5:**
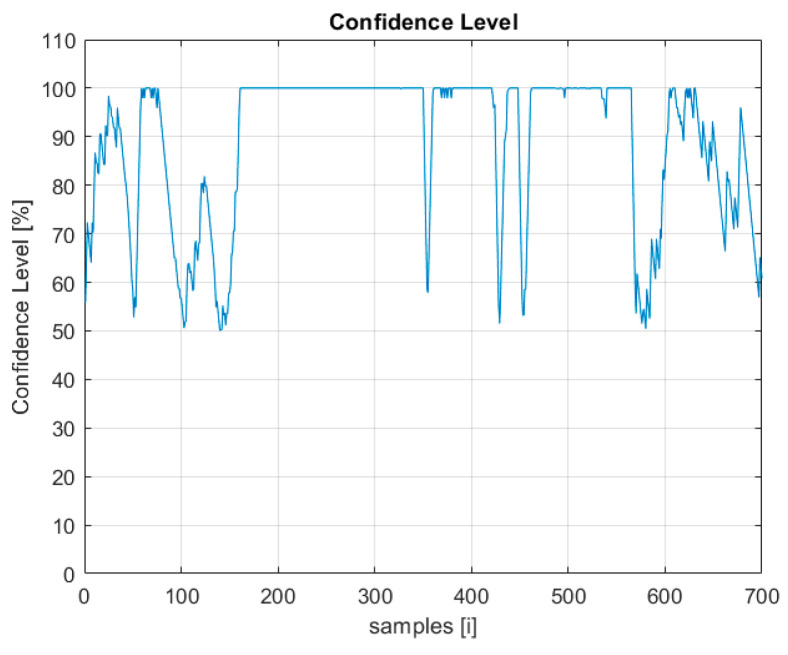
Radar detection confidence level plot.

Based on the flight logs stored, the time graphs of two indicators that will be used in the multisensory data fusion process are presented below: the probability of UAV detection (Figure 4) and the detection confidence level (Figure 5).

Information from several indicators, such as the detection probability (Figure 4) and the confidence level (Figure 5), may indicate detections classified with high probability as the UAV.

## 6. Sensory Fusion Concept

The flight logs contain information acquired by three types of sensors. Each of them has a different operating principle, and thus is a source of broad-spectrum data that complement each other. Moreover, each aforementioned information source bears some uncertainty and inaccuracy; therefore, the fusion of the information provided by all these sources makes it possible to improve the certainty of detection. There are several known data fusion methods based on, e.g., the Kalman filter, complementary filter, weighted function or prediction methods. The most common method of multisource data fusion is based on the Kalman filter concept [28]. Such an approach fits well in the situations when the dynamics of individual data sources vary significantly, which is not the issue of the study presented. The performed in-depth data analysis focused our investigation on data fusion through their conditional complementarity depending on the current conditions, imposed by the type of the identified UAV and its equipment (e.g., the presence of the ADS-B system). Moreover, data selected to be fused are the result of multistage analysis and extraction. For this reason, the application of the Kalman filter-based approach, which was nevertheless considered, was ultimately abandoned at the early stage of the project development. The conditional complementary filtration, which is the second reasonable methodology for multi-source data fusion, was used instead. The data analysis carried out in the previous section allowed for the formulation of several observations:

- Each sensor works asynchronously in an event-based way;
- Data provided by the AeroScope and EchoGuard radar systems contain interference in the form of short duration pulses of high amplitude;
- The ADS-B system provides information about all the aircrafts in the airspace which are within the range of the ADS-B receiver, both manned and unmanned;
- None of these sensors allows a complete detection, recognition and identification procedure;
- The desired effect can be achieved by fusing data from several sensors on the basis of intelligent information complementarity.

**Figure 6:**
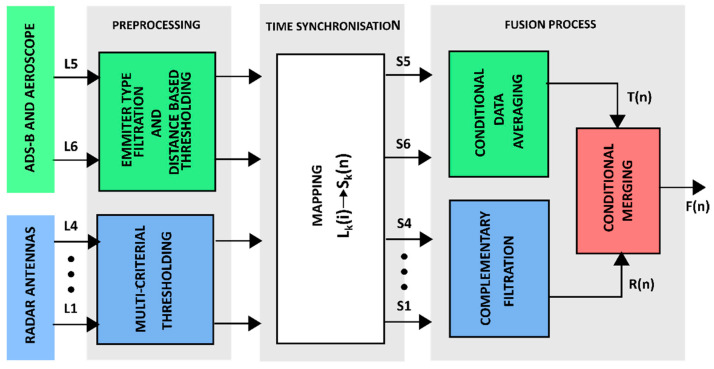
Diagram illustrating sensory fusion concept.

Figure 6 illustrates the concept of the data fusion process. As can be seen, this process is performed sequentially, through three stages. There are six independent information sources at the process input: ADS-B and AEROSCOPE transponders as well as four ECHOGUARD radar antennas. Since the data coming from transponders have different features than those provided by radar, they are processed independently. At the first pre-processing stage, outliers are detected and removed from the data sets. In the next phase, the data are synchronized in relation to the system initialization moment, with the given sampling period.

In the last stage synchronized data are fused in two steps. In the first one, independently, detections registered by transponders are merged using an averaging operation, while detections recorded by the four radar antennas are fused with the complementary filter. After this, these two independent channels are combined by the conditional merging.

### 6.1. Data Pre-Processing

Since it is much easier to analyse and process distance-based indices in the Cartesian space, all data on the location of detected objects are converted to the common Cartesian coordinate system, fixed to the centre of the radar station and oriented as follows: North-East-Up. Transponder data are converted from geographical coordinate space (lon-lat-alt). Radar readings are measured in a Cartesian frame fixed to individual radar antennas, and these data also have to be converted into the common coordinates frame.

As was mentioned above, the first step of the procedure consists of removing bad samples which are not going to be used in the fusion process. Since the data packages emitted by transponders have a relatively long spatial range, the receivers which are part of the system described can record information coming from many, often very distant, flying objects. So, the first operation which has to be performed is removing these detections using the distance-based thresholding. The ADS-B sensor can receive data broadcast by large aircrafts which are out of the scope of the system described. Therefore, in the first stage of signal processing, AV emitter type filtration was used. This means that from among all the aircraft identified by the system, only the data related to unmanned aerial vehicles (UAVs) should be extracted. In this case, a dedicated conditional filtering was used in which the *‘PingDetectionemitterType = 14’* parameter was used to extract the UAV. This parameter defines the category of the aircraft, and the value of ‘14’ unambiguously determines the unmanned aerial vehicle.

Taking into account the radar, the distance-based thresholding is also the first reasonable criterion of removing outlying detections. However, the radar software algorithms mark each detection with two additional tags which are useful in further data analysis. The first one, which is named *UAV Probability*, ranges from 0 to 1 and describes the certainty that the object detected belongs to the UAV class. The second one is the *Confidence Level*, which takes the values from the range of [0–100] and reflects the confidence that this detection is not measurement noise. In the pre-processing, high values of these tags are used to narrow down the set of analysed data.

### 6.2. Data Synchronisation

The multi-sensory fusion method outlined so far is based on the assumption that all data provided by particular sensors of the system are synchronous. Such an assumption is necessary to analyse spatial relations between detected objects. This means that for each instance n of discrete time, data sets determining detections collected at the same time can be distinguished: $S_{k}\left(n\right),\;k=1,\ldots ,K$, where n is the index of the given sensor. In real systems, such as the system described in this paper, both moments of detections and moments of recording them are event-related. This means that after classifying the given measurement (reading) as a detection of a UAV by the sensor algorithm, it is stored in the log-register with the time-stamp of the given sensor. The time-stamp is related to the particular sensor’s clock. Therefore, the first stage of the data fusion process is data synchronisation. For each instance n of discrete time, the following mapping has to be made:

(4) $$L_{k}\left(i\right)\to S_{k}\left(n\right)$$

where *i* denotes the detection index recorded in the log-register of the *k*th sensor, whereas *n* defines subsequent moments in time, $t_{n}=n\Delta t,\;n=1,2,\ldots$, discretised with the sampling period Δt. It is performed by labelling elements of original sets $L_{k}$ with indices belonging to the given set $S_{k}\left(n\right)$. Each set contains detections acquired in the period of time tϵ<(n−1)Δt, nΔt>. In particular, these sets may be empty, which means there was not any detection in the given time interval. Each record of data provided by individual sensors is given a unique identification number. In each sample period *n*, multiple detections of the same ID can be registered. During the process of synchronising, these detections have to be merged into a single one. This operation can be performed by using various operators. In the case of this work, the averaging operation was applied.

### 6.3. ADS-B and AEROSCOPE Data Fusion

**Figure 7:**
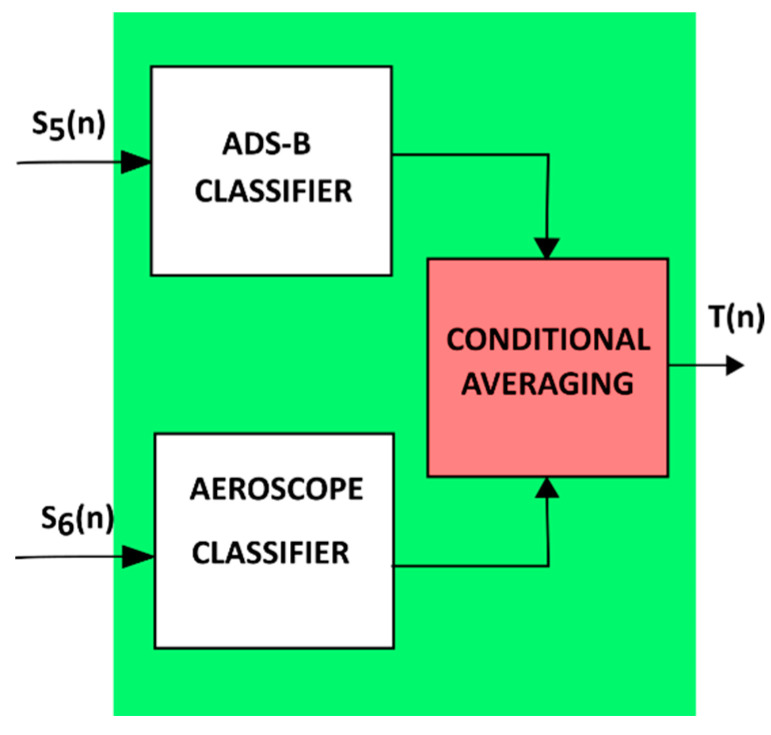
Transponders data fusion idea.

The idea of a complementary filter is well known from inertial measurement units IMU, the task of which is to estimate the orientation of the UAV based on measurements from independent sensors, characterised by the complementation of information in the frequency domain [29,30,31,32,33,34]. While analysing the sensory data acquired by the system, it was noticed that within some areas, the detections provided by multiple sensors complement each other. Detections of the ADS-B and AeroScope systems are very similar, provided that the observed UAV is a DJI platform. In cases where the observed area is violated by a UAV of another manufacturer, then the ADS-B system detections will complement the radar indications. Taking into account the above observations, a data fusion based on conditional complementarity was proposed (Figure 7).

Considering the filter’s activity in the assumed time interval, it checks first whether the ADS-B and AeroScope systems have detected the presence of the UAV. If both sensors registered detections, then these detections are averaged in the considered time interval. If the detection appeared only in the data doming from the ADS-B, it means that the detected UAV is not a DJI drone, and in this case, detections provided by the ADS-B are taken as the result of the fusion. However, when the ADS-B and AEROSCOPE systems do not show UAV activity, and the detections appear only in the radar readings, then most likely there is a UAV of a different manufacturer than DJI in the observed area, with neither an ADS-B nor an AEROSCOPE transponder installed. Therefore, the output data of this part of the system take the form of a conditional sum:

(5) $$T\left(n\right)=S_{5}\left(n\right)\cup S_{6}\left(n\right)\cup {\bar{S}}_{5,6}\left(n\right)$$

where ${\bar{S}}_{5,6}\left(n\right)$ is the average of the elements of data sets $S_{5}\;$and $S_{6}$.

### 6.4. Radar Data Fusion

Radar enables detection, localisation and motion parameter measurements of an object moving within its range. The detection range of a radar is dependent on its sensitivity and the spatial configuration of its antennas. Also, environmental conditions taken together with the aforementioned factors imply that the same object tracked by multiple antennas may be detected with different accuracy and certainty. For example, the certainty of an object detection moving at the limit of the sensor’s range is usually lower than the object localised in the center of the detection area.

**Figure 8:**
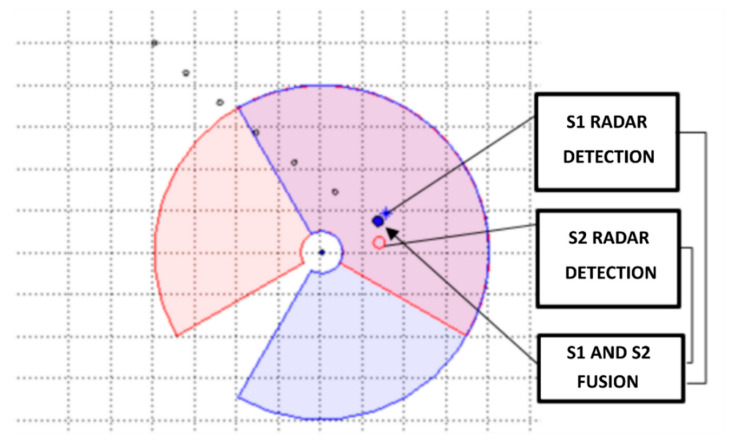
Geometrical interpretation of the case of multiple radar detection of the same object.

So, having information about detections coming from multiple radar antennas monitoring the same observation area, it is usually possible to improve detection accuracy and certainty. Figure 8 gives an interpretation of this case. There is a moving object within the range of two antennas of the radar station (*S*_1_ and *S*_2_; their detection areas are plotted with red and blue colors). The real localisation of the object in subsequent moments in time is marked with black circles, while its current location in the *n*th moment is marked with a blue circle. Detections of this object registered by antennas *S*_1_ and *S*_2_ are marked with the red circle and the blue star, respectively. The result of the fusion of information provided by these two sensors is plotted with the black circle.

In the case of multiple detections, the problem is determining which of the detections represent the same object, then how to make their fusion. In this paper, we applied the distance-based clustering and complementary filtration with weighting factors calculated as functions of the certainty factor.

Let us assume that the given area is monitored by four radar antennas of different spatial configurations. Let us also assume that *M* unidentified objects are moving within this area. The set of detections captured by the *k*th antenna in the time *n* is denoted as

(6) $$S_{k}\left(n\right)=\left\{d_{i}^{k}\right\},\;i=1,2,\ldots ,M,\;k=1\ldots 4$$

where

(7) $$d_{i}^{k}=\left(P_{i}^{k},c_{i}^{k}\right),\;c_{i}^{k}\in \left[0,1\right]$$

The $P_{i}^{k}$ denotes the *i*th detection of the *k*th antenna described in Cartesian spatial coordinates, while $c_{i}^{k}$ is the detection certainty factor estimated by the internal algorithm of the radar system. 

The first stage of the fusion procedure is identifying the given object among all detections captured by particular antennas. It is performed by determining the similarity (in the sense of the metric used) of elements belonging to the sets $S_{1}\ldots S_{4}$. The similarity is related to the spatial proximity of the elements; therefore, the most convenient and intuitive metric is the Euclidean one, which was used in the described approach.

Two elements captured by different sensors at the same moment *n* in time are considered to be similar if the distance between them is smaller than some threshold value:

(8) $$\left|d_{i}^{k}-d_{j}^{l}\right|<THR,\;k,l=1,2,\ldots ,4,\;i\in \left[1,M_{k}\right],\;j\in \left[1,M_{j}\right]$$

This threshold depends on many issues, such as the spatial resolution of the sensors, and is tuned experimentally. The result of the similarity checking process is the similarity matrix:

(9) $$A_{s}=\left\{a_{n,m}\right\},\;n\in \left[0,N_{s}\right],\;m\in \left[1,4\right]$$

where *m is* equal to the number of sensors and *n* is the number of pairs of elements classified as similar, respectively. For example, the matrix row of elements [1 0 2 0] means that the first element of the set $S_{1}$ is similar to the second element of the set $S_{3}$, which in turn implies that antennas 1 and 3 detected the same physical object.

The next step of the data aggregation procedure is merging the detections of the same object captured by multiple radar antennas, indicated by the similarity matrix. Each row of this matrix indicates a pair of detections which are close enough in the sense of the threshold applied. In the approach presented, the complementary weighted average was used for merging the similar detections. The weighting factors are computed the way providing that the detection captured with a higher certainty factor also has a higher influence on the fusion result. Let us assume two detections classified as similar are given by

(10) $$d_{i}^{k}=\left(P_{i}^{k},c_{i}^{k}\right)\;\mathrm{and}\;d_{j}^{l}=\left(P_{j}^{l},c_{j}^{l}\right)$$

The fusion $\left(\tilde{P},\tilde{c}\right)$ of these two elements is calculated as

(11) $$\tilde{P}=w_{1}P_{i}^{k}+w_{2}P_{j}^{l}$$

(12) $$\tilde{c}=\max\left(P_{i}^{k},P_{j}^{l}\right)$$

The weighting factors are computed complementarily as the functions of certainty factors:

(13) $$\{\begin{array}{c} w_{1}=0.5\left(1+\frac{1}{1+e^{-\alpha \left(c_{i}^{k}-THR\right)}}\right),\;w_{2}=1-w_{1}\;\;\;\;\;\;for\;\;c_{i}^{k}>c_{j}^{l}\\ w_{1}=0.5\left(1+\frac{1}{1+e^{-\alpha \left(c_{j}^{l}-THR\right)}}\right),\;w_{2}=1-w_{1}\;\;\;\;\;\;for\;\;c_{i}^{k}\leq c_{j}^{l} \end{array}$$

After this stage of the fusion process, for the *n*th moment in time, the following vector of detections is obtained:

(14) $$R\left(n\right)=\left[{\tilde{d}}_{1}\left(n\right),{\tilde{d}}_{2}\left(n\right),\ldots ,{\tilde{d}}_{M_{n}}\left(n\right)\right],\;\;\;M_{n}\leq M$$

### 6.5. Final Fusion

At this stage of the process, detections coming from two separate information channels (transponders and radar) have to be merged into one. Let us denote the set containing fused readings coming from transponders ADS-B and AeroScope, recorded in the sampling period *n* as *T*(*n*). On the other hand, there is a set of radar detections fused recorded in the same sampling period—*R*(*n*). The fusion of these stets is performed using the following conditional scheme:

(15) $$F\left(n\right)=\{\begin{array}{c} T\left(n\right)\;\;\;\;\;\;\;\;\;\;\;\;if\;T\left(n\right)\neq \emptyset \;\cap \;R\left(n\right)=\emptyset \;\\ R\left(n\right)\;\;\;\;\;\;\;\;\;\;\;\;if\;T\left(n\right)=\emptyset \;\cap R\;\left(n\right)\neq \emptyset \;\\ \tilde{F}\left(n\right)\;\;\;\;\;\;\;\;\;\;\;\;\;\;\;\;\;\;\;\;\;\;\;\;\;\;\;\;\;\;\;\;\;\;\;\;\;\;\;\;\;\;otherwise \end{array}$$

The first two cases of this scheme are obvious and will not be commented upon. Let us take a closer look at the third one, describing the situation where both the transponders and the radar registered detections. In such a case, there is a need to distinguish two cases. First, when readings coming from the transponders and the radar refer to the same object, they have to be merged. Second, when detections registered by the radar and the transponder receivers are disjointed, both detections are stored in the files of the system records. Hence, the following conditional procedure is performed:

(16) $$\tilde{F}\left(n\right)=\{\begin{array}{c} T_{i}\left(n\right)\cup R_{j}\left(n\right)\;\;\;if\;\;\left|T_{i}\left(n\right)-R_{j}\left(n\right)\right|>THR\\ T_{i}\left(n\right)\;\;\;\;\;\;\;\;\;\;\;\;\;\;\;\;\;\;if\;\;\left|T_{i}\left(n\right)-R_{j}\left(n\right)\right|\leq THR \end{array}$$

So, if the distance between detections $T_{i}\left(n\right)$ and *R*_*j*_(*n*) is greater than the assumed threshold *THR*, both detections are saved in the records. Otherwise, there is a high possibility that two detections refer to the same object. In this case, detection $T_{i}\left(n\right)$ coming from the transponder is kept, since it is more reliable.

### 6.6. Detection Identification and Tracking

At the last stage of the process of retrieving information upon violation of the observed airspace, identification of the detection and tracking the identified object must be provided. This is the most important information from the end user perspective, and performing these operations simultaneously is highly complex. Of course, all the preceding data processing is absolutely necessary to perform the last stage, and this must be highlighted. The identification of the objects detected by individual radar antennas is based on signatures given to them by the radar software algorithms. Similarly, data sent by transponders contain their own signatures identifying the objects. Further, while merging the detections, this information is lost. Therefore, it is very important to combine new incoming detections with those previously registered. In other words, the following similarity must be found:

(17) $$F_{i}\left(n\right)\sim F_{j}\left(n-1\right),\;\;i=1,2,\ldots ,\overset{=}{\;F}\left(n\right),\;\;j=1,2,\ldots ,\overset{=}{\;F}\left(n-1\right),$$

where the time index *n* denotes the current detection while *n –* 1 *is* the previously obtained one. 

One of the possible solutions to the aforementioned problem is using the predictive approach. The method proposed consists of comparing the detection’s prediction to the current detection, using the Euclidean metric:

(18) $$F_{i}\left(n\right)\sim {\hat{F}}_{j}\left(n\right),\;\;i=1,2,\ldots ,\overset{=}{\;F}\left(n\right),\;\;j=1,2,\ldots ,\overset{=}{\;F}\left(n-1\right),\;$$

where ${\hat{F}}_{j}\left(n\right)$ denotes the detected objects’s location prediction calculated for the current moment *n*. The prediction is the function of the past readings:

(19) $${\hat{F}}_{j}\left(n\right)=f\left(F_{j}\left(n\right),F_{j}\left(n-1\right),\ldots ,F_{j}\left(n-H\right)\right),$$

where *H* is the number of past readings taken for calculating the prediction. Another option that allows the location prediction of the considered object to be obtained is using information on its velocity. Since the radar provides estimates of the detection’s velocities measured in relation to three axes, it is easy to find the prediction of the object location using these data:

(20) $${\hat{F}}_{j}\left(n\right)=f\left(F_{j}\left(n-1\right),v_{x,j}\left(n-1\right),\;v_{y,j}\left(n-1\right),v_{z,j}\left(n-1\right)\right)$$

One more possibility of finding similarity between current and past detections is comparing them directly using the Euclidean metric.

In all the aforementioned options, if the distance between the current and previous detection are recognized as similar, the current detection is given to the identifier of the previous one. Otherwise, the current one is treated as a new detection and is given a new ID.

## 7. Results

The purpose of the task was to validate by simulation the algorithm developed for detecting single, as well as multiple, UAVs. As part of this task, the results generated by the algorithm implemented were verified by comparing them to the data obtained as a result of the planned experiment. Before commencing validation tests, measurement data were acquired by the sensory system during test flights. The tests covered:

- Single UAV flight—DJI Matrice 600;
- Simultaneous flight of two UAVs—DJI Matrice 600 and DJI Mavic 2.

In the case of using the DJI Matrice 600 platform, a full set of measurement data was provided, which includes ADS-B, AeroScope and radar (four sector antennas). In turn, using the DJI Mavic platform, AeroScope and radar data were provided.

### 7.1. Single UAV Detection

**Figure 9:**
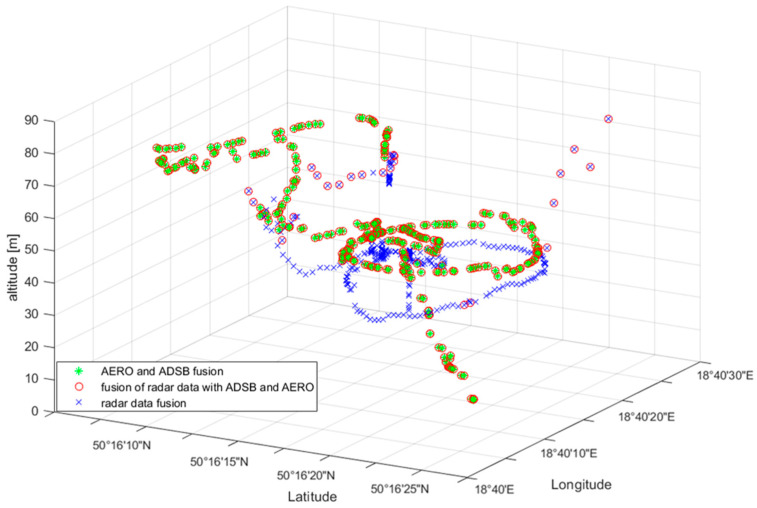
UAV flight path registered by various sensors after the information fusion.

**Figure 10:**
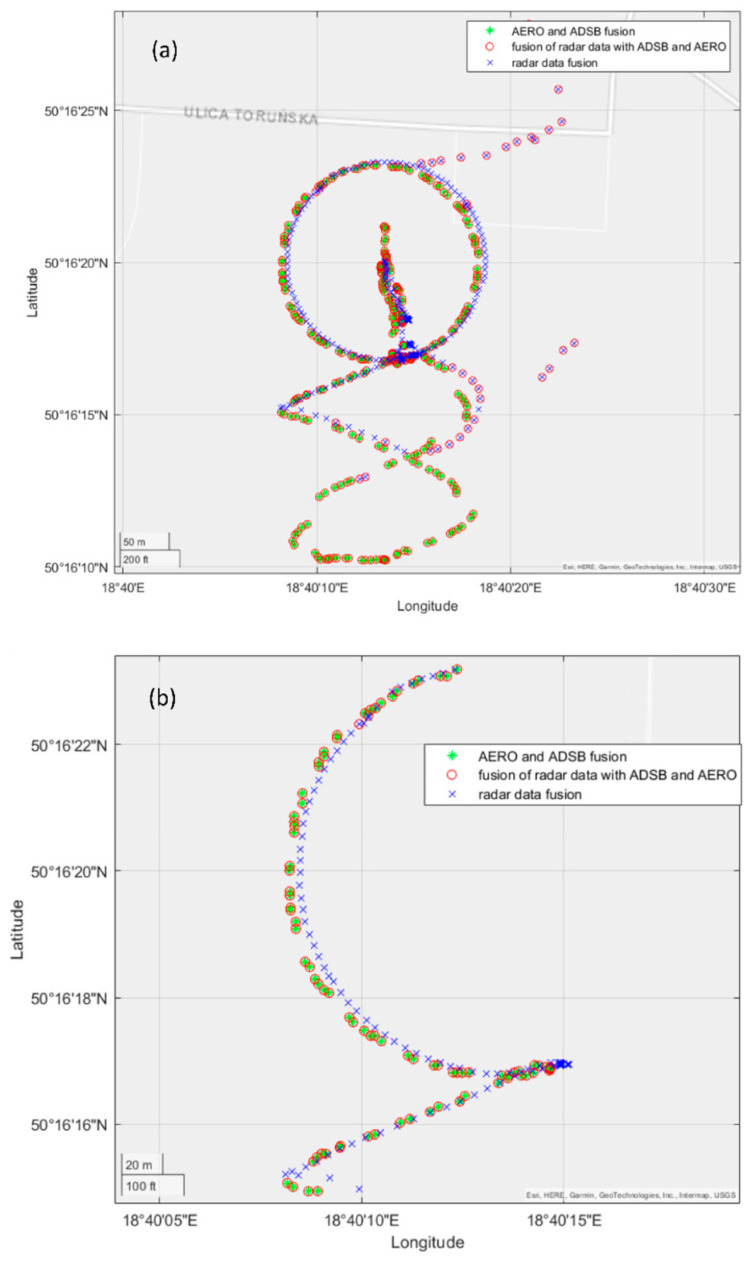
UAV flight path presented in 2D geographical coordinates (**a**) and its enlarged part (**b**).

The first test consisted of performing an operator-controlled flight of the UAV of type DJI Matrice 600 within the monitored area of restricted airspace. As was mentioned before, this drone was equipped with both ADS-B and AREOSCOPE transponders. The radar antennas were mounted on the 8 m high mast. During the experiment, data coming from all sensors were saved. After the flight, the data acquired were post-processed. Time synchronisation with the sampling period equal to 1 [s] was forced. Figure 9 shows the result of the fusion procedure described in the previous section, presented using a 3D graph. Detections registered by the radar system, after filtering and merging data coming from four antennas, are marked with the blue crosses. In the case of radar data fusion, additional detections appeared, clearly visible on the 2D projection of the flight trajectory (Figure 10), which were the result of the assumed threshold values of complementary filtration. The threshold values used in the filtration process were: PUAV = 0.7 and CL = 80%.

**Figure 11:**
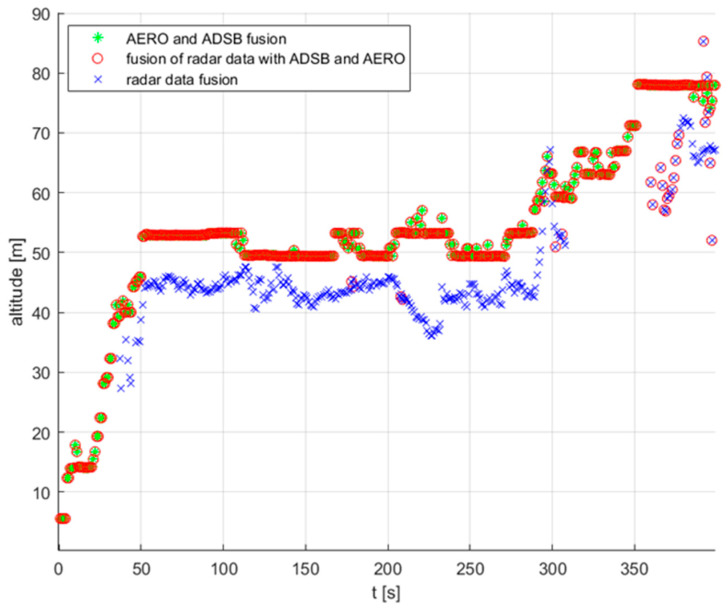
UAV flight altitude plot.

On the other hand, the UAV flight path registered by transponder systems ADS-B and AREOSCOPE after the fusion process is plotted with a red circle. As one can notice by looking at this picture, there is a slight offset between the transponders’ and radar’s data regarding the flight altitude measurements. This phenomenon is illustrated more precisely in Figure 11. The reason for such a discrepancy in the presented results comes from the fact that the assembly offsets of the radar antennas were not measured precisely enough. Finally, a complete fusion of the transponder and radar data are presented in Figure 9 with green stars. As was explained in the previous section, data coming from transponders, registered by a GPS system, are considered as more reliable than radar measurements. Therefore, in cases where there are both radar and transponder data in the given sampling period, the fusion results in neglecting the radar readings.

Figure 10 shows the 2D interpretation of the aforementioned fusion aspects: the entire flight path (a) and the enlarged part of the flight path for the time interval of 200–300 [s].

### 7.2. Multiple UAV Detection

**Figure 12:**
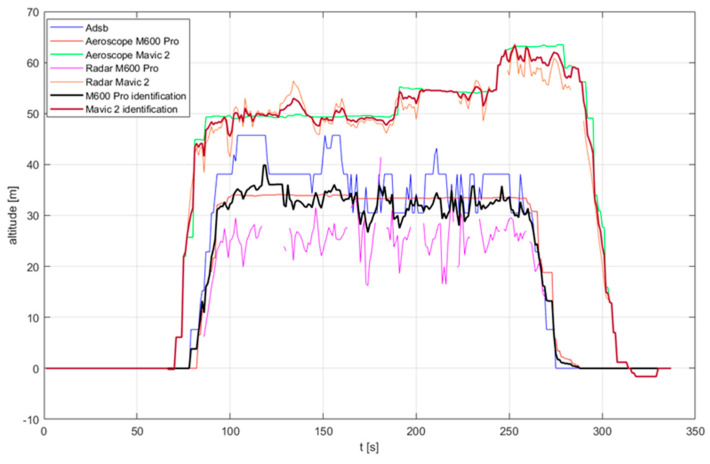
Multiple UAV flight altitudes–indirect detections and identifications.

System tests were performed comprehensively and followed the system integration stage. At this stage of the project, the end-to-end test was performed. The test scenario assumed the simultaneous flights of two drones—the Mavic 2 and Matrice 600 Pro. The flight altitude of the first one was about 50 [m], whereas the second drone was flying at the altitude of 30 [m]. The radar antennas assembly configuration was the same as in the experiment described in Section 7.1. Also, the notation used for marking individual stages of data processing is the same. Again, as in the previous scenario, data acquired from transponders were used as the reference to the radar measurements. This time, let us start the results analysis from observing the recorded altitude data, presented in Figure 12.

Let us consider the flight of the M600 drone. Analysing the AREOSCOPE data, we can see an almost flat, very precise record of the flight altitude at the level of 32 [m]. Taking into account the ADS-B readings, also mounted onboard the M600, the readings are not so accurate and oscillations of the amplitude of about 10 [m] can be observed. As for the radar detections of this object, a slight offset of about −5 [m] can be seen. Finally, after the fusion of data from these three sources, the M600 flight altitude path was extracted (black line). On the other hand, analysing the Mavic 2 drone flight, we can conclude that the data describing the altitude registered by radar and AEROSCOPE are more similar to each other. As in the first case, the fusion procedure gave a quite reliable flight path identification (red line).

**Figure 13:**
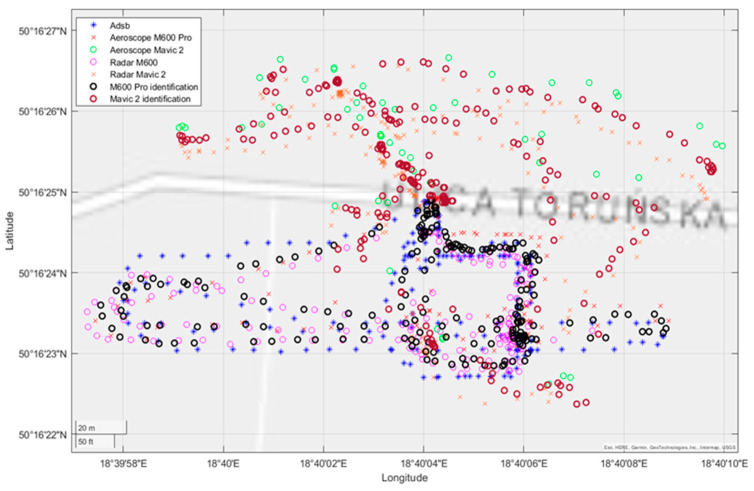
Flight trajectory in 2D space—indirect detections and identifications.

Figure 13 shows detected objects on the longitude–latitude plane. Readings from transponders are marked as explained by the picture legend. As mentioned before, they are considered as a reference to the data fusion results. We can observe a slight offset between the ADS-B and AEROSCOPE data identifying the M600 drone. Looking at the radar fusion results, it can be noticed that both drones were detected, identified and tracked.

**Figure 14:**
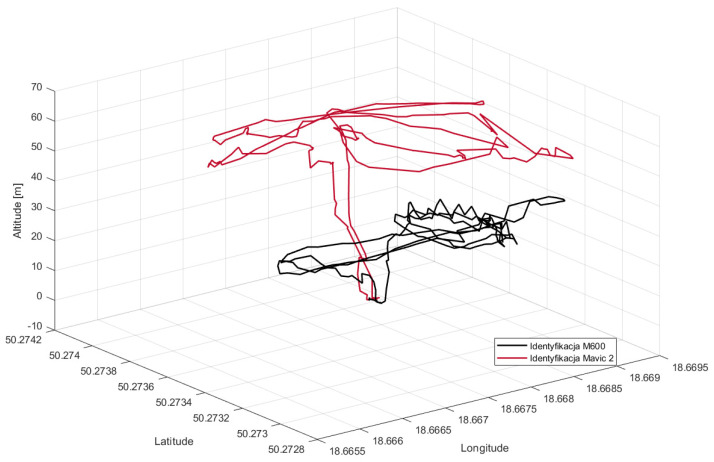
Flight trajectory in 3D space−final result of data fusion.

Finally, the fusion of all informational channels results in registering precise tracks of objects violating the observation area. This is shown even better in the last figure as a 3D plot (Figure 14). Since transponders’ data contain information about the type of the UAV, these objects were identified as two drones: M600 and Mavic2. Of course, due to only having radar readings, it is not possible to perform high level identification. Nevertheless, as is shown in Figure 13, the radar was able to identify two separate tracks of UAVs flying within the monitored space.

## 8. Conclusions

This paper concerned the problem of the detection of unmanned aerial vehicles violating restricted airspace. At the outset, the review of available market solutions in the field of anti-drone systems and existing detection technologies made the reader aware of the possibilities of counteracting unmanned aerial vehicles. The performed analysis also showed the shortcomings of the existing anti-drone systems and indicated new research directions. In the next stage, the data provided by the sensory system during field tests in real conditions was analysed. The algorithm of fusing data acquired by multiple sensors, enabling the UAV detection, identification and recognition in 3D space, was proposed. The developed detection system includes the following three subsystems: pre-processing, time synchronisation and data fusion, which is based on complementary conditional filtering. The efficiency of the proposed solution was tested on the basis of rapid prototyping in the MATLAB simulation environment using data from the real sensory system obtained during controlled UAV flights.

It must be concluded that if a UAV entering the range of the monitoring system is equipped with an ADS-B or AEROSCOPE transponder, there is no problem with detecting and identifying the aerial vehicle. Unfortunately, the most likely scenario is that the UAV violating the restricted airspace will intentionally not be equipped with such devices. Then, the system detection abilities are based on radar readings. The radar used in this project is very sensitive and is able to detect small objects—either miniature drones or other objects. This property of the sensor raises further problems with data interpretation. Usually, the radar captures many more objects than the ones being subject to the observation. In this paper, we proposed multistage filtration to reject the false detections. The efficiency of the proposed approach was proved by multiple tests. The next crucial problem addressed in this paper was the data fusion of detections registered by multiple radar antennas with overlapping fields of view. The complementary filter-based fusion, presented in Section 6.3, solved this problem in a satisfactory way. The next issue is the identification and tracking of the detected object using radar readings. In the fusion process, unique signatures of detections given to the objects by the radar software disappear and new identifications have to be given to the fused data. In the next phases of the system operation, this identification must be tracked to provide continuous observation of the identified object. This problem was solved in this project by using the predictive clustering method. In this case, satisfactory results were obtained as well. However, though the operational abilities of the monitoring system based on the presented methodology were in general satisfactory, further works on improving its efficiency are required. The most crucial issues that must be revisited are the radar data fusion in terms of overlapping observation areas and the detection tracking. Applying more sophisticated methods of providing a more reliable prediction of the tracked object seems to be a favourable starting point for the method’s improvement.

## Glossary

*ICAO*	aircraft type code
*lat*_*i*_	latitude indicated by the *i*th sensor
*long*_*i*_	longitude indicated by the *i*th sensor
*h*	altitude
*y*	heading
V	flight velocity
$V_{xy}$	horizontal velocity
$V_{z}$	vertical velocity
*ET*	category of the aircraft emitting the signal
*d*	distance of the UAV from the sensory system
*DT*	UAV type
*Did*	UAV identifier
*P*_UAV_	probability of UAV detection
*CL*	confidence level
*RCS*	radar track RCS (radar cross-section)
*az*	estimated azimuth
*el*	estimated elevation
*R*	estimated distance between UAV and radar
*x*, *y*, *z*	estimated UAV coordinates relative to the radar (in Cartesian coordinate system)
$V_{x},\;V_{y},\;V_{z}$	velocity components of UAV in the *x*, *y* and *z* axes, respectively
$S_{k}\left(n\right)$	data sets determining detections collected in the same time
*k*	sensor index
*n*	subsequent moments in time $t_{n}=n\Delta t,\;n=1,2,\ldots$
Δt	sampling period
$L_{k}\left(i\right)$	mapped detection set
*i*	detection index recorded in the log-register
*T*(*n*)	set of fused detections from ADS-B and AREOSCOPE
$P_{i}^{k}$	*i*th detection of *k*th antena
$c_{i}^{k}$	detection certainty factor
$A_{s}$	detection similarity matrix
*R*(*n*)	set of fused radar detections
F(n)	fusion of *T*(*n*) and *R*(*n*) sets
